# Next-generation sequencing yields the complete mitochondrial genome of the Shangrila hot-spring snakes (*Thermophis shangrila*; Reptilia: Colubridae)

**DOI:** 10.1080/23802359.2017.1331330

**Published:** 2017-05-30

**Authors:** Wei Wu, De-Chun Jiang, Feng-Hui Sun

**Affiliations:** a Engineering Laboratory of Prevention and Control of Veterinary Drug Residues in Animal Derived Food, Chengdu Medical College, Chengdu, China;; b Chengdu Institute of Biology, Chinese Academy of Sciences, Chengdu, China

**Keywords:** Mitogenome, next-generation sequencing, *Thermophis shangrila*

## Abstract

In this study, we sequenced the complete mitochondrial genome of *Thermophis shangrila* by using the next-generation sequencing technique. The total length of the mitogenome was 17,407 bp, which was composed of 13 protein coding genes, two rRNA genes (12s and 16s rRNA), 22 tRNA genes, and two control regions (CRI and CRII). The base composition was 32.6% for A, 23.9% for T, 30.0% for C, and 13.5% for G. We added a fragment about 150 bp in length at control region I, which Peng et al. failed to obtain using Sanger dideoxy sequencing.

The hot-spring keel-back (*Thermophis*), which contains only three species (*T. zhaoermii*; *T. baileyi*; *T. shangrila*), achieves the world’s highest altitude distribution (over 4000 m) among all snakes (Huang et al. 2009; He et al. 2010; Alex et al. 2016). *Thermophis shangrila* was identified as a new species of *Thermophis* by Peng et al. (2014). In this study, we sequenced the complete mitochondrial genome of *T. shangrila* by using the next-generation sequencing (NGS).

*T. shangrila* were collected from Tianshengqiao Shangri-La, Yunnan province, China. Voucher specimens were deposited at CIB herpetological museum, Chengdu Institute of Biology with the number CIBLJT20150813. The total genomic DNA was extracted from the fresh muscle tissue. We got mitochondrial genome directly from NGS data, which was generated from total genomic DNA extracts (Hahn et al. 2013). Then, we downloaded RefSeq (mitogenome of *Thermophis* zhaoermii) from Organelle Genome Resources database at NCBI (https://www.ncbi.nlm.nih.gov/genome/organelle/) and assembled *de novo* mitogenome using MITObim (Paszkiewicz & Studholme 2010). Gene structure was predicted by Mitos (Bernt et al. 2013). The RefSeq was also used to correct the mitogenomic structure manually. The base composition was calculated by MEGA5 (Tamura et al. 2001). The tRNA genes were scanned by tRNAscan-SE (Lowe & Eddy 1997).

The complete mitogenome sequence together with gene annotations were deposited in the GenBank under the accession number MF066951. The total length of the *T. shangrila* mitochondrial genome was 17,407 bp, which was slightly longer than 17,327 bp as reported by Peng et al. (2016). The base composition was 32.6% for A, 23.9% for T, 30.0% for C, and 13.5% for G. The mitogenome we sequenced contained 13 protein coding genes, two rRNA genes (12s and 16s rRNA), 22 tRNA genes, and two control regions (CRI and CRII), which were similar to the other reported snakes (He et al. 2010). As for sequence features of protein-coding genes, start codons were ATG for COXII, COXIII, ATP8, ATP6, ND4L, ND4, ND5, and CYTB; ATA for ND1, ND2, and ND3; and GTG for the remaining one (COXI). TAA was the stop codon for ATP8, ATP6, ND4L, and ND5. AGG was the stop codon for ND4. Furthermore, the length of 22 tRNA genes ranged from 57 to 74 bp, of which the shortest one was tRNASer (AGY) and the longest was tRNAAsn. The sequence length of 12s and 16s rRNA were 925 bp and 1479 bp, respectively, which were separated by tRNAVal. As reported by Peng et al. (2016), CRI was 1175 bp, which was longer than CRII, as CRII was 1026 bp.

For convincing the mitochondrial DNA sequences, a maximum likelihood (ML) phylogenetic tree was re-constructed in the program RAxML-HPC (Stamatakis 2006) using alignment of coding regions of 11 mitochondrial genomes of Colubridae (Figure 1). Phylogenetic analysis showed that *T. shangrila* was located within the lineage of Colubridae and was sister species of *T. zhaoermii* as previously described (Sun et al. 2011; Alex et al. 2016).

**Figure 1.**
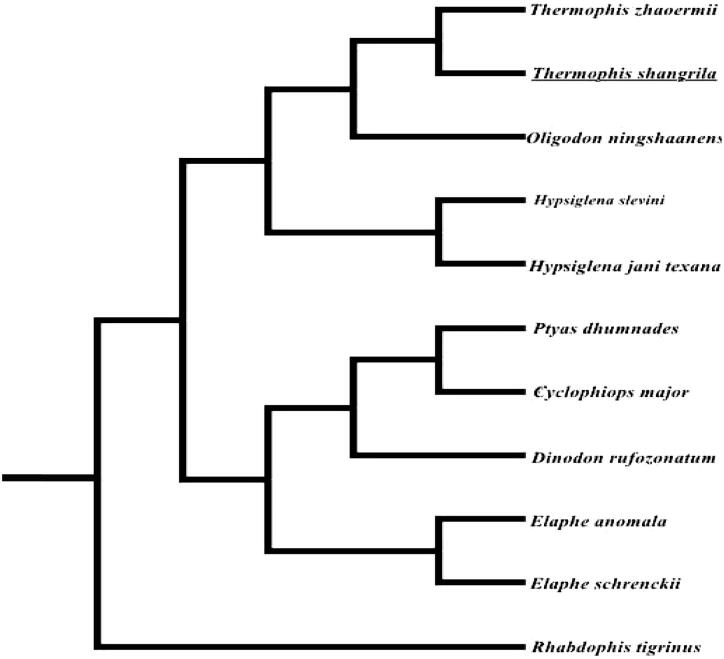
The ML tree of 11 species from Colubridae was constructed based on the protein-coding genes except ND6. The GenBank accession number is as follows: *Dinodon rufozonatum* (KF148622), *Rhabdophis tigrinus* (NC030210), *Elaphe schrenckii* (NC027605), *Elaphe anomala* (NC027001), *Elaphe schrenckii* (NC027605), *Cyclophiops major* (NC028048), *Ptyas dhumnades* (KF148621), *Oligodon ningshaanensis* (KJ719252), *Hypsiglena jani texana* (EU728592), *Hypsiglena slevini* (EU728584), *Thermophis zhaoermii* (GQ166168).

In this study, we revealed the complete mitochondrial sequence of *T. shangrila* and sequenced the fragment from 3809 to 3961 bp, which failed to be shown by Peng et al. (2016). We hope that this determination about *T. shangrila* mitochondrial genome will provide useful resources for better understanding the evolution of genus *Thermophis* and the whole Colubridae.
